# Plant-Based Scaffolds Modify Cellular Response to Drug and Radiation Exposure Compared to Standard Cell Culture Models

**DOI:** 10.3389/fbioe.2020.00932

**Published:** 2020-08-07

**Authors:** Jerome Lacombe, Ashlee F. Harris, Ryan Zenhausern, Sophia Karsunsky, Frederic Zenhausern

**Affiliations:** ^1^Center for Applied NanoBioscience and Medicine, College of Medicine Phoenix, University of Arizona, Phoenix, AZ, United States; ^2^Department of Biomedical Engineering, College of Engineering, University of Arizona, Tucson, AZ, United States; ^3^School of Pharmaceutical Sciences, University of Geneva, Geneva, Switzerland

**Keywords:** tissue engineering, decellularization, plant-based scaffold, stiffness, YAP/TAZ pathway, radiation

## Abstract

Plant-based scaffolds present many advantages over a variety of biomaterials. Recent studies explored their potential to be repopulated with human cells and thus highlight a growing interest for their use in tissue engineering or for biomedical applications. However, it is still unclear if these *in vitro* plant-based scaffolds can modify cell phenotype or affect cellular response to external stimuli. Here, we report the characterization of the mechano-regulation of melanoma SK-MEL-28 and prostate PC3 cells seeded on decellularized spinach leaves scaffolds, compared to cells deposited on standard rigid cell culture substrate, as well as their response to drug and radiation treatment. The results showed that YAP/TAZ signaling was downregulated, cellular morphology altered and proliferation rate decreased when cells were cultured on leaf scaffold. Interestingly, cell culture on vegetal scaffold also affected cellular response to external stress. Thus, SK-MEL-28 cells phenotype is modified leading to a decrease in MITF activity and drug resistance, while PC3 cells showed altered gene expression and radiation response. These findings shed lights on the decellularization of vegetal materials to provide substrates that can be repopulated with human cells to better reproduce a soft tissue microenvironment. However, these complex scaffolds mediate changes in cell behavior and in order to exploit the capability of matching physical properties of the various plant scaffolds to diverse physiological functionalities of cells and human tissue constructs, additional studies are required to better characterize physical and biochemical cell-substrate interactions.

## Introduction

The use of porous three-dimensional (3D) scaffolds to provide a suitable environment for the generation of tissues and organs is vital for tissue engineering applications, or for the exploration of novel cellular models for biomedical research. For these purposes, several biomaterials have been explored including ceramics, metals, bioactive glasses, animal-derived tissues, polymers, etc. (Place et al., 2009), but only recently plants and plant-based polymers have emerged as relevant biomaterials. First, the plant-based scaffolds have many practical advantages including the apparent ease with which they can be made and manipulated; they are quite pliable and can be easily cut, fashioned, rolled or stacked to form a range of different sizes and shapes (Iravani and Varma, 2019). They are also renewable, easy to mass produce and are relatively inexpensive. Second, from a physical and architectural perspectives, plant tissues have promising properties, including high surface area, interconnected porosity, natural vascular networks, various range of stiffness and mechanical properties, and excellent water transport and retention (Adamski et al., 2018). Finally, from a biochemical perceptive, plant-based scaffolds are mainly made of cellulose, a biocompatible and non-immunogenic material that allows for cell adhesion (Hickey and Pelling, 2019) and even demonstrated pro-angiogenic function *in vivo* (Modulevsky et al., 2016).

Several years ago, a study demonstrated that apple tissue could be decellularized, and the remaining cellulose scaffold could be employed for *in vitro* cell culture, demonstrating that naturally derived cellulose scaffolds offer a complementary approach to existing techniques for the *in vitro* culture of mammalian cells in a 3D environment (Modulevsky et al., 2014). More recently, a study showed the great potential of decellularized spinach leaves to model the cardiac environment by recellularizing both the inner vascular network of the plant with human endothelial cells and the surface of the leaf with cardiomyocytes showing that a multitude of plant-derived cellulose scaffolds are suitable *in vitro* (Gershlak et al., 2017). Many different cell types have been used to repopulate decellularized plant-derived scaffolds, including human endothelial cells (Gershlak et al., 2017; Dikici et al., 2019), human dermal fibroblasts (Fontana et al., 2017; Dikici et al., 2019), mouse fibroblasts (Modulevsky et al., 2014; James et al., 2020), mouse myoblasts (Modulevsky et al., 2014), human cervical cell lines (Modulevsky et al., 2014), human aortic smooth muscle cells (James et al., 2020), mesenchymal stem cells (Fontana et al., 2017; Gershlak et al., 2017; James et al., 2020) and stem cells derived cardiomyocytes (Gershlak et al., 2017), suggesting that cellulose scaffolds can attach either cell lines or primary cells.

These proof-of-concept studies demonstrated the biocompatibility of vegetal scaffolds with mammalian cells which can adhere, proliferate and stay at least partially functional. However, whether the cellular behavior is affected by such scaffolds, for example after external stress exposure, is still uncharacterized and needs to be further investigated. This is a critical validation, if plant-based materials have to become more popular in tissue engineering, or to be used as an alternative to the standard cell culture model.

Consequently, we assessed in this study the cellular response to the plant-based scaffolds-induced stress by comparing the regulation of mechanotransduction pathways of cells seeded on decellularized spinach leaves compared to cells seeded on conventional cell culture substrates such as standard tissue culture polystyrene (TCPS) flasks or glass coverslips. We later investigated whether vegetal scaffolds could modify cell phenotype and drug response in melanoma cells or radiation response in prostate cancer cells compared to standard two-dimensional cell culture models.

## Materials and Methods

### Decellularization of Plant Tissues

Plant material, including baby spinach leaves (*Spinacia oleracea*), hybrid cherry tomato plant (*Solanum lycopersicum*), aquatic plant (*Echinodorus grisebachii*) A. Borealis (*Kalanchoe fedtschenkoi variegati*) and lucky bamboo (*Dracaena sanderiana*) were purchased from the local store. To initiate decellularization, spinach, tomato and aquatic leaves were cannulated through the petiole (base of the stem) with a 26-gauge needle and secured with heat shrink tubing. A. Borealis and lucky bamboo were not cannulated but directly soaked in the different solutions on an orbital shaker. The wax cuticle protecting the leaf was removed with three cycles of alternating washes with hexanes and phosphate-buffered saline (PBS). The prepared leaves were connected to gravity bags filled with the different solutions. Decellularization by chemical treatment started with perfusion of a solution of 1% sodium dodecyl sulfate (SDS) in deionized (DI) water for 2 days, followed by a solution of 10% sodium chlorite and 0.1% Triton-X 100 in DI water for 2 days. The leaves were then flushed with DI water for an additional 48 h. Leaves were stored in DI water at four degrees Celsius (°C) until ready for use.

### DNA/Protein Quantification

Leaves were first placed in centrifuge tubes and then were disrupted after freezing in liquid nitrogen by using a pestle. Resulting powder was transferred to a microcentrifuge tube for DNA and protein quantification. DNA content of fresh and decellularized leaves was extracted using DNeasy Plant Mini Kit (Qiagen) following manufacturer recommendation, while proteins were extracted by radioimmunoprecipitation assay (RIPA) (Pierce RIPA buffer, Thermo Fisher Scientific) for 30 min on ice. DNA content was quantified by reading absorbance at 260 nm and protein content was quantified using microBCA protein assay kit (Thermo Fisher Scientific). Both absorbances were measured using Epoch microplate spectrophotometer (BioTek Instruments).

### Human Cell Culture and Seeding on Leaf Scaffold

Prostate cancer cells (PC3) and melanoma cells (SK-MEL-28) were obtained from ATCC (CRL-1435 and HTB-72, respectively). PC3 cells were cultured in Roswell Park Memorial Institute 1640 medium and SK-MEL-28 in Minimum Essential Medium, both supplemented with 10% fetal bovine serum (FBS) and 1% Penicillin/Streptomycin (P/S). Prior to recellularization, spinach leaf scaffolds were first sterilized using a UV Stratalinker 2400 (Stratagene) for 30 min. Leaf structures were then functionalized with collagen and fibronectin proteins. Briefly, scaffolds were incubated in 50 μg/ml of collagen I (A1048301, Thermo Scientific) in 20 mM acetic acid solution for 4 h, followed by two washes in PBS and a final wash in complete medium. Leaves were then incubated in 10 μg/ml fibronectin (F0895, Sigma-Aldrich) for 24 h followed by three washes in complete medium. Finally, treated leaves were cut into small pieces and fit to the bottom of untreated multiple well plates. To prevent the leaf from freely floating in the well and to facilitate cell confinement to the leaf area, hollow inserts were placed on the top of the leaf. Cell attachment was promoted for different periods of time, depending on the assay, at 37°C with 5% CO_2_ atmosphere.

### Atomic Force Microscopy (AFM)-Based Imaging and Force Mapping

Scanning images were acquired with a Nanosurf LensAFM (Nanosurf, Switzerland) coupled with an upright Nikon Eclipse E800 optical microscope (Nikon, Japan). The SCM-PIC cantilevers (Bruker) were 405–495 μm long, 45–55 μm wide, and 1.5–2.5 μm thick. Their spring constant was 0.2 N/m and their resonant frequency in an aqueous solution was 13 kHz. The images were acquired with 512 points by 512 lines. The scanning range was 87.5 μm × 87.5 μm and the scan time per line was 2 s. All AFM observations were performed at room temperature (24–26°C) and acquired with Nanosurf easyScan 2 3.8.0 software.

For force measurement, the Young’s Modulus (YM) of the leaf scaffolds were determined using force spectroscopy mode at liquid interface (in air for lucky bamboo) with a Nanosurf Flex-Bio AFM System (Nanosurf, Switzerland). Gold coated qp-BioAC cantilevers (Nanosensors), 80 μm in length, 30 μm in width, 400 nm thick, with a nominal force constant of 0.06 N/m and a resonance frequency of 30 kHz were used for leaves measurement while Multi75GD-G (NanoAndMore), 225 μm in length, 28 μm in width, 3 μm thick, with a nominal force constant of 3 N/m and a resonance frequency of 75 kHz were used for the lucky bamboo. The samples were first fixed to a glass slide with vacuum grease and mounted on a magnetic AFM stage at room temperature (24–26°C). First, the spring constant of the cantilever was calibrated by using the thermal tune method on a cleaned and stiff surface (Mica) and then force curves were measured. For each plant, force maps were recorded at three different positions on the same leaf for at least 3 different leaves. Each force map contained 64 force curves (8 × 8 lines per frame) over an area of typically 10 μm. Force maps were processed with the C3000 Nanosurf software and the YM was extracted using AtomicJ 1.7.2 software, assuming a Poisson’s ratio of 0.5 (Hermanowicz et al., 2014).

### 3D Surface Mapping

Topographical images were directly taken from fresh, decellularized and recellularized leaves using a tactile sensor pad imaged with a GelSight, Inc., Benchtop System. The deformable gel elastomer pad (Medium-Firm, 20180524-001) was pressed onto each leaf. Six photographs were acquired by a standard DSLR camera (Canon Rebel T3i) with 18 megapixels resolved with a 5X lens (Canon MP-E 65 mm 1–5X Macro Lens). Each image is taken from a different angle illuminated with LED lighting. The images represent a 4.5 mm × 3.0 mm area and are combined with GelSight software (GSCapture) to generate a textural map of the leaf surface.

### MTT Cell Viability Assay

A modified MTT experiment was developed from CellTiter 96 Non-Radioactive Cell Proliferation Assay kit (Promega). Treated leaves were first cut into pieces to fit the bottom of an untreated 96-well plate and cells seeded at 16,000 cells/cm^2^. After 24 h, to avoid measuring any residual cells attached to the bottom of the plate, leaves with attached cells were transferred to a new plate. In parallel, cells were also directly seeded into a treated 96-well plate at a concentration of 5,000 cells/well (∼16,000 cells/cm^2^). The medium was refreshed every 72 h. Tetrazolium component was added at day 1, 3, 5, and 7 and absorbance of formazan product was measured at 570 nm by using Epoch microplate spectrophotometer (Biotek Instruments).

For the drug response assay, withaferin A (WFA) from the aerial parts of aeroponically grown *W. somnifera* was used and characterized as previously described (Lacombe et al., 2019). WFA was added at concentrations ranging from 0.156 to 40 μM. DMSO (0.8%) served as vehicle and control. Cells were incubated for 72 h prior to the addition of the tetrazolium. The concentration of drugs that resulted in 50% of cell death (IC_50_) was determined from dose-response curve by using PRISM 7.0 (GraphPad100 Software, San Diego, CA, United States). Experiments were repeated three times, and data represented as the mean of quadruplicate wells ± SEM.

### Immunoblotting

For protein extraction, recellularized leaf scaffolds and cells in TCPS at 80% confluency were first washed three times with PBS to remove excess of cell culture medium. The leaf scaffold was then immerged in RIPA buffer for 30 min on ice with regular vortex steps while RIPA was directly added into the TCPS for 15 min on ice before cell scrapping. Cell lysates were then centrifuged at maximal speed, supernatants were collected and protein contents were quantified using microBCA protein assay kit (Thermo Fisher Scientific). Absorbance was read using an Epoch microplate spectrophotometer (BioTek Instruments). Samples (5 μg total protein) were mixed with Laemmli sample buffer (Bio-Rad) and heated at 95°C for 5 min. Samples were then loaded on graded pre-cast polyacrylamide gels (4–20% mini PROTEAN TGX gels, Bio-Rad), separated based on size by electrophoresis (90 V for 2 h) and transferred (300 mA for 90 min) to PVDF membranes (Bio-Rad). Membranes were then blocked with PBS plus 0.1% Tween-20 containing 5% dried non-fat milk at room temperature for 1 h. Blots were then incubated at 4°C overnight with primary antibodies against Yorkie-homolog/yes-associated protein – 1 (YAP) (sc101199; Santa Cruz; 1/200) and transcriptional coactivator with PDZ-binding motif (TAZ) (#4883; Cell Signaling; 1/1,000). Glyceraldehyde 3-phosphate dehydrogenase (GAPDH) (sc365062; Santa Cruz; 1/200) was used as the control. After five washes (5 min/each) in PBS plus Tween-20, membranes were incubated with anti-mouse HRP-conjugated antibody (1/10,000; Jackson ImmunoResearch Laboratories) or anti-rabbit HRP-conjugated antibody (1/5,000; Jackson ImmunoResearch Laboratories) at room temperature for 1 h. After five additional washes, membranes were developed with ECL substrate (Clarity Western, Bio-Rad) and imaged using a GBox Chemi doc system (XX6, Syngene). Full size image is provided in Supplementary Figure S5. Band intensities were quantified by using ImageJ and differences between experimental conditions were analyzed using the Mann-Whitney test.

### Real-Time Quantitative Reverse Transcription PCR (qRT-PCR)

Cells were seeded at 80,000 cells/cm^2^ on functionalized leaves and TCPS. After 72 h (and 6 h after irradiation for DNA damage signaling pathway assay), cells were lysed and RNA was extracted using RNAspin Mini kit (GE Healthcare, 25-0500-71) according to the manufacturer’s recommendation. RNA quantification was performed using Epoch microplate spectrophotometer (BioTek Instruments). RNA was stored at −80°C until further use.

For mechanotransduction studies, one microgram of total RNA was first used to generate cDNA using a QuantiTect Reverse Transcriptase kit (Qiagen, #205310) according to manufacturer’s instructions. Briefly, the reaction was first incubated at 42°C for 2 min to eliminate residual genomic DNA and then placed immediately on ice. After addition of the reverse transcription mix, reaction was then placed at 42°C for 15 additional minutes and stopped by incubation at 95°C for 3 min before proceeding to PCR. QuantiFast SYBR Green PCR reactions (Qiagen, #204054) were carried out on a 96-well plate format using a Stratagene Mx30005P (Agilent Technologies, Inc.). The cDNA was added to the plate along with the RT Mastermix containing 2X QuantiFast SYBR Green PCR Master Mix, RNase-free water and primers (1 μM): ANKRD1 (F: AGAACTGTGCTGGGAAGACG; R: GCC ATGCCTTCAAAATGCCA), CTGF (F: AGGAGTGGGTGTGT GACGA; R: CCAGGCAGTTGGCTCTAATC), GAPDH (F: CT CCTGCACCACCAACTGCT; R: GGGCCATCCACAGTCTTC TG) MITF (F: GTTGCCTGTCTCGGGAAACT; R: TACACGC TGTGAGCTCCCTT), MLANA (F: GGGAGTCTTACTGCTCA TCGG; R: TCAAACCCTTCTTGTGGGCA), SOX10 (F: GGA GGCTGCTGAACGAAAGT; R: GGGCGCTCTTGTAGTGGG), TAZ (F: GGACCAAGTACATGAACCACC; R: TGCAGGACT GGTGATTGGAC), TYR (F: CGAGTCGGATCTGGTCATGG; R: GACACAGCAAGCTCACAAGC) and YAP (F: CCCTCG TTTTGCCATGAACC; R: GTTGCTGCTGGTTGGAGTTG). Cycling parameters were 5 min at 95°C for initial activation, followed by 40 cycles of denaturation at 95°C for 10 s and combined annealing and extension at 60°C for 30 s. Melting curves were automatically generated, ranging step-wise from 60 to 95°C. Data were collected by MxPro qPCR Software (Agilent). Values were normalized with glyceraldehyde-3-phosphate dehydrogenase (GAPDH) and analyzed according to delta Ct method (Livak and Schmittgen, 2001).

For DNA damage signaling pathway assay, qRT-PCR was performed using RT_2_ Profiler PCR Array Human DNA Damage Signaling Pathway (Qiagen, #330231). Three hundred nanograms of total RNA were first used to generate cDNA with RT^2^ First Strand Kit (Qiagen) according to manufacturer’s instructions. Briefly, the reaction was first incubated at 42°C for 5 min to eliminate residual genomic DNA and then placed 1 min on ice. After addition of the reverse transcription mix, reaction was then placed at 42°C for 15 additional minutes and stopped by incubation at 95°C for 5 min before proceeding to PCR. The cDNA was then added to the RT_2_ Profiler PCR 96-well plate along with the RT Mastermix containing 2X RT2 SYBR Green. Cycling parameters were 10 min at 95°C for initial denaturation, followed by 40 cycles of denaturation at 95°C for 15 s and annealing and extension at 60°C for 1 min. Melting curves and data were collected as previously described. Values were normalized with actin, beta (ACTB); beta-2-microglobulin (B2M); glyceraldehyde-3-phosphate dehydrogenase (GAPDH); hypoxanthine phosphoribosyltransferase 1 (HPRT1) and ribosomal protein, large, P0 (RPLP0) and analyzed according to delta Ct method (Livak and Schmittgen, 2001). Gene expression were considered to be significantly modified if the associated *p*-values were less than 0.05 and fold changes (FC) was greater than 1.5 or less than 0.66.

### Scanning Electron Microscopy (SEM)

Cells were seeded at 12,000 cells/cm^2^ on functionalized leaves and glass coverslip. After 72 h, they were fixed with 2.5% glutaraldehyde in 1X PBS overnight at 4°C. Samples were dehydrated in increasing concentrations of ethanol (50, 70, 85, 95, 95, 100, 100%) for 1 h each and left overnight in 100% ethanol at 4°C. Samples were left to dry on pin stub mounts (12.7 mm × 8 mm, Ted Pella) and sputter coated with gold (1 0 nm) prior imaging to the Eyring Materials Center at Arizona State University with a SEM-FEG XL30 (FEI).

### Irradiation

All irradiation was performed using a cabinet X-ray machine (X-RAD 320, Precision X-Ray Inc., North Branford, CT) at 320 kVp and 12.5 mA with a 2 mm Al filter. Dose-rate was 3 Gy/min. The source-to-axis distance was 42 cm. The beam was calibrated using a UNIDOS E PTW T10010 electrometer and TN30013 ionization chamber, with measurement done in air, for a 15 cm × 15 cm field size.

### Immunofluorescence Staining and Microscopy

Cells were seeded at 12,000 cells/cm^2^ on functionalized leaves or glass coverslip. After 72 h, cells were fixed with 4% paraformaldehyde (PFA) in PBS for 15 min and permeabilized with 0.1% Triton X-100/PBS for 5 min. For γ-H2AX foci formation assay, cells were fixed and permeabilized at 1, 6, and 24 h after 2 Gy-irradiation. Then, cells were blocked for 30 min at room temperature in 1% bovin serum albumin (BSA)/PBS and immunostained with antibodies diluted in 1% BSA/PBS to γH2AX Ser139 (JBW301; Upstate Cell Signaling; 1/800), alpha-tubulin (sc5286; Santa Cruz; 1/150), beta-catenin (sc7963; Santa Cruz; 1/100), YAP (sc-101199; Santa Cruz; 1/50), and Ki-67 (ab15580; AbCam; 1/1000) followed by Cy3-conjugated anti-mouse IgG (115-165-062, Jackson ImmunoResearch, 1/1000) and Alexa Fluor 647-conjugated anti-rabbit IgG (111-605-045, Jackson ImmunoResearch, 1/500) or Alexa Fluo 647-conjugated Phalloidin (#A22287, Life Technologies, 1/40) and counterstained with DAPI. Images were obtained using a Zeiss Axio Imager M2 epifluorescent microscope and were acquired with a Zeiss AxioCam MRm camera using ZEN 4.5 software at the Biomedical Imaging Core Facility at the UA College of Medicine – Phoenix.

### Clonogenic Assay

Cells were cultured both on spinach leaf or in TCPS for 3 days. Then, cells were harvested by trypsin, counted, seeded in triplicate into 6-well plates at appropriate cell densities and immediately irradiated at 0, 2, 4, and 6 Gy. Then, cells were cultured for 14 days, and they were fixed with 6.0% glutaraldehyde and stained with 0.5% crystal violet. Colonies with more than 50 cells were counted and surviving fractions were calculated on the basis of the plating efficiencies of corresponding non-irradiated cells.

### Statistical Analysis

All statistical tests and graphs were performed with GraphPad Prism version 7.00 for Windows (GraphPad Software Inc., La Jolla, CA, United States). ImageJ software was used to quantify γH2AX and 53BP1 foci, immunoblot bands intensities and cell spreading areas. All results are presented as mean ± SEM. Statistical comparisons were made by using Student’s *t*-test or Mann-Whitney test if data distribution did not pass normality test. All differences were considered statistically significant when *p* < 0.05.

## Results

The spinach leaves have been decellularized following standard serial chemical treatment (Fontana et al., 2017; Gershlak et al., 2017; Dikici et al., 2019). After 7 days of treatment, leaves lost chlorophyll and appeared fully translucent (Supplementary Figure S1A) suggesting that plant material have been successfully removed from the native structure. In order to assess the efficiency of this decellularization process, protein and DNA content was then quantified. Decellularized leaves contained significantly less DNA (Supplementary Figure S1B) and protein (Supplementary Figure S1C) compared to fresh leaves, below the minimal requirement to consider a tissue as decellularized (Crapo et al., 2011). In addition, AFM-based imaging showed that plant structures, such as stoma and veins, are more visible on the decellularized leaves compared to fresh leaves (Supplementary Figure S1D). Finally, measurement of Young’s modulus by using force-distance curves-based AFM showed that the decellularized scaffold is much less rigid than the native leaf (139.4 ± 5 MPa vs 21.8 ± 3.3 kPa) (Supplementary Figure S1E). Altogether, these data suggested that decellularization treatment was effective to remove all vegetal content.

To ensure that cells can adhere, prostate cancer cells (PC3) were then seeded on decellularized spinach scaffolds. Since mechanical and biochemical properties of the extracellular matrix (ECM) regulate cell morphology, adhesion, proliferation, communication and tissue formation (Kular et al., 2014; Theocharis et al., 2016; Vogel, 2018), spinach scaffolds were first functionalized with a combination of collagen and fibronectin treatment in order to improve cell attachment and better mimic the presence of ECM. After 24 h incubation, fluorescence microscopy imaging was performed confirming that PC3 cells are present on the leaf surface (Supplementary Figure S2A). We also performed a larger scale optical 3D leaf topography and texture imaging by using a GelSight tactile sensor (Johnson et al., 2011). The photometric results showed an apparent vasculature on decellularized leaf masked by presence of plant cells on fresh leaf and, in a lesser degree, by human cells on the recellularized scaffolds (Supplementary Figure S2B). In addition, fluorescence imaging showed that cells were able to create cell-cell junctions, such as adherens junctions, when visualized by the expression of β-catenin (Supplementary Figure S2C). Finally, well-formed mitotic spindles were visible in cells showing different phases of mitosis and thus demonstrating that cells are alive and able to divide on the leaf scaffold (Supplementary Figure S2D). All together, these results demonstrated that cells were able to attach onto the functionalized spinach scaffold and were able to proliferate and form a tissue-like layer, as demonstrated previously (Gershlak et al., 2017; Dikici et al., 2019).

### YAP/TAZ Signaling Pathway Is Downregulated in Cells Cultured on Spinach Leaf Decellularized Scaffolds

**FIGURE 1:**
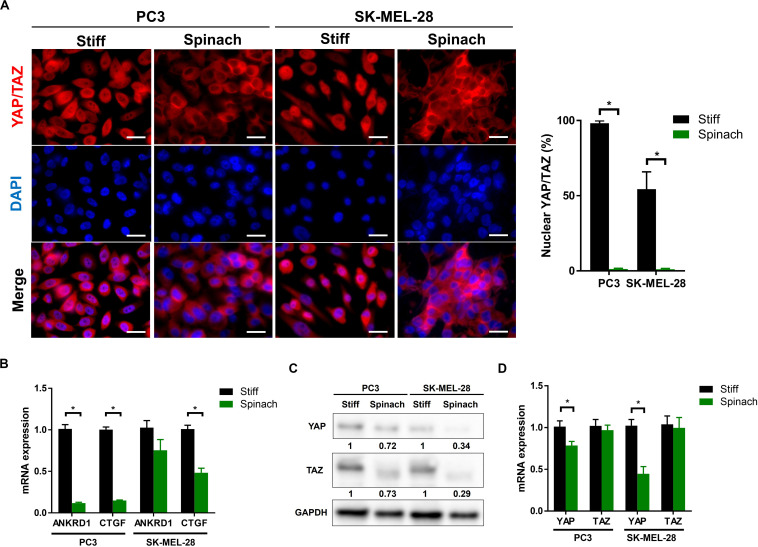
YAP/TAZ signaling pathway was downregulated in cells cultured on spinach leaf decellularized scaffolds. **(A)** Immunofluorescence images of YAP/TAZ and nuclei (DAPI) in PC3 and SK-MEL-28 cells seeded on coverslip and spinach scaffold for 3 days. Scale bars = 15 μm. Graphs indicate the percentage of cells with nuclear YAP/TAZ (*n* = 3, *p < 0.05; Student *t*-test). **(B)** Quantitative RT-PCR analysis in PC3 and SK-MEL-28 cells to measure YAP and TAZ mRNA levels. Cells were grown on TCPS or spinach scaffold for 3 days. Data were normalized to expression on TCPS and indicated as mean ± SEM (*n* = 3, *p < 0.05; Student *t*-test). **(C)** Immunoblotting of YAP and TAZ in PC3 and SK-MEL-28 cells seeded on TCPS and spinach scaffolds. Bands intensities were quantified using ImageJ and normalized with GAPDH. Numbers represent the expression level compared to TCPS of three independent experiments. **(D)** Quantitative RT-PCR for YAP/TAZ target genes (CTGF and ANKRD1) in PC3 and SK-MEL-28 cells. Cells were grown on TCPS or spinach scaffold for 3 days. Data were normalized to expression on TCPS and indicated as mean ± SEM (*n* = 3, *p < 0.05; Student *t*-test).

Since decellularized spinach scaffold is relatively soft (21.8 kPa) and substrate stiffness directly influences cellular mechano-regulation (Andalib et al., 2016; Cantini et al., 2020), we first investigated if the emerging model of Yorkie-homolog YAP (Yes-associated protein) and TAZ (transcriptional coactivator with PDZ-binding motif, also known as WWTR1) pathways as a master regulator of mechanotransduction response (Totaro et al., 2018), could be modified when cells were seeded on vegetal scaffold compared to stiff substrates, such as TCPS or glass coverslips. Therefore, endogenous YAP/TAZ subcellular localization was first assessed by immunofluorescence in PC3 and melanoma SK-MEL-28 cells seeded on spinach leaf and stiff substrate. Results showed that YAP/TAZ were clearly nuclear on stiff substrate but became predominantly cytoplasmic on cells cultured on spinach leaf scaffold (Figure 1A). In addition, mRNA expression of YAP was significantly decreased in PC3 and SK-MEL-28 cell lines (*p* = 0.031 and *p* = 0.0001, respectively) when cultured on spinach scaffolds while mRNA TAZ expression was not modified (Figure 1B). YAP and TAZ proteins were also under-expressed in both cell lines when cultured on spinach scaffolds compared to stiff substrate (Figure 1C). Finally, the measurement of the expression of two YAP/TAZ regulated genes, ANKRD1 and CTGF, showed that ANKRD1 was significantly downregulated in PC3 cells and CTGF was significantly downregulated in both cell lines seeded on leaf scaffold compared to stiff substrate (Figure 1D). Altogether, these results suggested that YAP/TAZ pathway was downregulated in cells cultured on leaf scaffold.

### Cell Culture on Leaf Scaffold Induces Cell Morphology Changes

**FIGURE 2:**
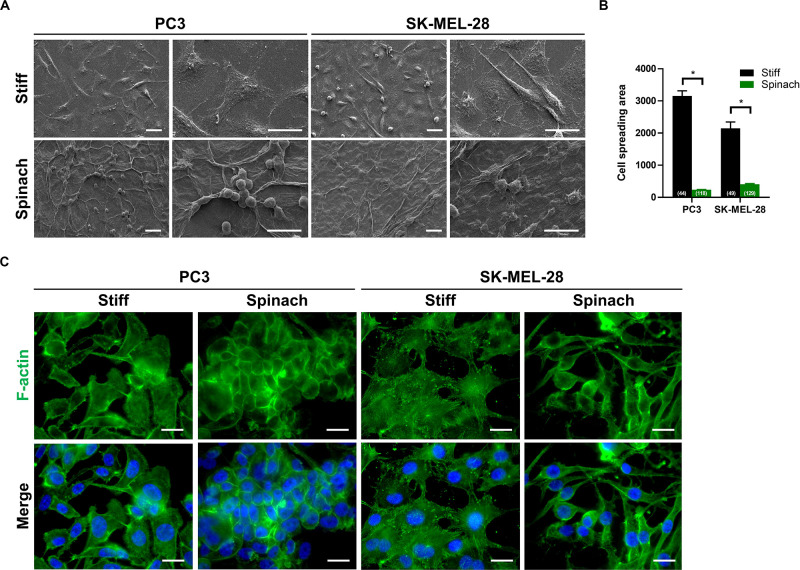
Cell culture on leaf scaffold induced cell morphology changes. **(A)** Representative SEM images of PC3 and SK-MEL-28 cells cultured on stiff substrate (coverslip) or spinach leaf scaffold for 3 days. The cell images were collected in three independent experiments (*n* = 3). Scale bars = 20 μm. **(B)** Histogram showing the changes of cell spreading area on stiff and leaf substrates and represented as mean ± SEM (*n* = 3, *p < 0.05; Mann-Whitney test). The numbers shown in parenthesis indicated cell numbers for statistics of cell spreading area examined in each case. **(C)** Immunofluorescence images of F-actin and nuclei (DAPI) in PC3 and SK-MEL-28 cells seeded on stiff substrate (coverslip) and spinach scaffold. Scale bars = 15 μm.

We then investigated whether the cellular morphology could be altered when cells were seeded on spinach scaffolds. Scanning electron microscopy revealed that both PC3 and SK-MEL-28 cells seeded on stiff substrate displayed an extended shape, with longer cell cilia, and a broader cell body compared to cells seeded on leaf scaffold that presented a round shape (Figure 2A). The cell spreading area was quantified and shown to be 92.4 and 81% reduction for PC3 and SK-MEL-28 cells, respectively, when they are cultured on leaf scaffold (238.7 and 408.2, respectively) compared to the stiff substrate (3154.3 and 2148.9, respectively) (Figure 2B). In addition, F-actin immunofluorescence confirmed this observation by revealing that cells maintain their round morphology with diffuse actin on leaf scaffold, whereas cells seeded on plastic substrate displayed a polygonal morphology with numerous F-actin stress fibers (Figure 2C).

### Proliferation Rate Is Decreased for Cells Seeded on Leaf Scaffold

**FIGURE 3:**
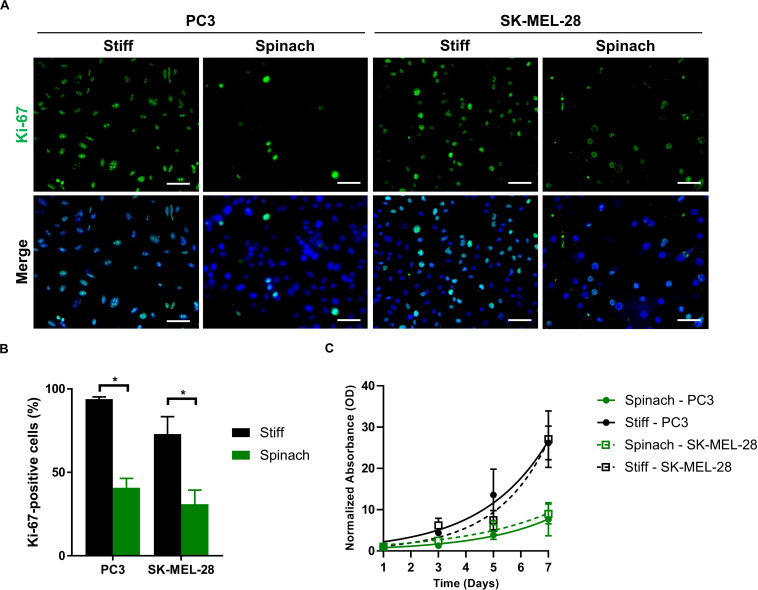
Proliferation rate was decreased for cells seeded on leaf scaffold. **(A)** Immunofluorescence images of Ki-67 and nuclei (DAPI) in PC3 and SK-MEL-28 cells seeded on stiff substrate (coverslip) and spinach scaffold. Scale bars = 60 μm. **(B)** Histogram showing the percentage of Ki-67-positive cells and represented as mean ± SEM (*n* = 3, *p < 0.05; Mann-Whitney test). **(C)** Cell proliferation of PC3 and SK-MEL-28 cells seeded for 7 days on stiff (TCPS) and spinach leaf substrate measured by modified MTT. Absorbance (570 nm) values were normalized from 100% at day 1 and analyzed using a non-linear regression using exponential growth curve. Data points represent the mean ± SEM (*n* = 3 with four replicates each).

The proliferation activity of PC3 and SK-MEL-28 cells on stiff and leaf substrates was then assessed by measuring Ki-67 staining. Immunofluorescence showed that Ki-67 expression was upregulated in cells cultured for 72 h on stiff substrate compared to leaf scaffold (Figure 3A). The number of Ki-67-positive cells was significantly lower for PC3 and SK-MEL-28 cells (*p* < 0.0001 and *p* = 0.003, respectively) when they are cultured on spinach leaf scaffold (Figure 3B), suggesting a potential higher proliferation rate of cells seeded on stiff substrate. To confirm this hypothesis, a modified MTT was then performed to assess proliferation of PC3 and SK-MEL-28 seeded on the leaf compared to a stiff substrate. Results showed that after 7 days, the normalized absorbance for PC3 and SK-MEL-28 cells grown on the stiff substrate was, respectively, 3.5-fold and three-fold higher compared to the values for cells grown on the spinach leaf scaffold (Figure 3C). Together these results showed that the cells growing on leaf scaffold had a lower proliferation rate than cells growing on a stiff substrate.

### Cell Culture on Leaf Scaffold Changes Melanoma SK-MEL-28 Cells Phenotype and Response to Drug Exposure

**FIGURE 4:**
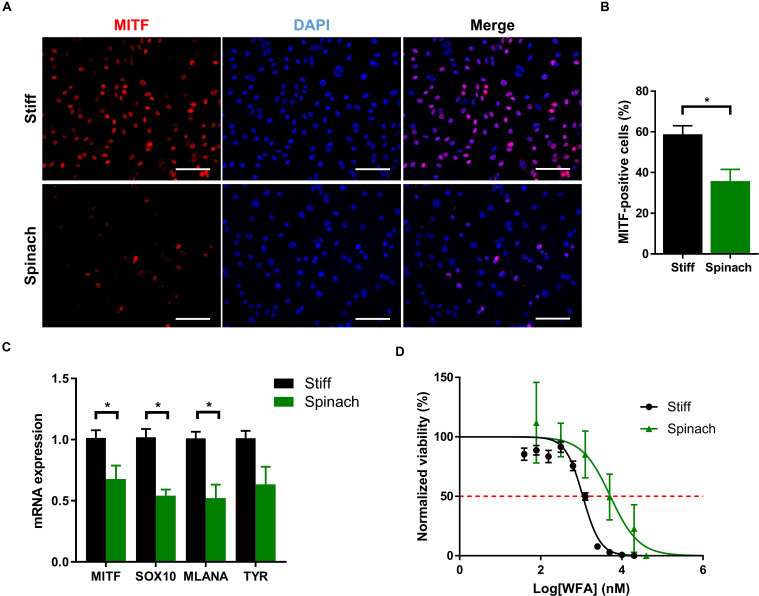
Cell culture on leaf scaffold decreased MITF expression and induced WFA resistance in melanoma SK-MEL-28 cells. **(A)** Immunofluorescence images of MITF and nuclei (DAPI) in SK-MEL-28 cells seeded on stiff substrate (coverslip) and spinach scaffold. Scale bars = 60 μm. **(B)** Histogram showing the percentage of MITF-positive cells and represented as mean ± SEM (*n* = 3, *p < 0.05; Student *t*-test). **(C)** Quantitative RT-PCR analysis in SK-MEL-28 cells to measure MITF and there MITF target genes (SOX10, MLANA, and TYR) mRNA levels. Cells were grown on TCPS or spinach scaffold for 3 days. Data were normalized to expression on TCPS and indicated as mean ± SEM (*n* = 3, *p < 0.05; Student *t*-test). **(D)** Effect of WFA treatment on SK-MEL-28 seeded on decellularized spinach leaves or TCPS assessed by modified MTT. Cells were exposed to either vehicle (DMSO) or a large range of concentrations (0.039 to 20 μM) of WFA for 72 h. Absorbance (570 nm) values were normalized from 100% in DMSO control and analyzed using a non-linear regression using dose response curve fitting [log (inhibition) vs normalized response (variable slope)]. Red dotted line intercepts dose-response curves at 50% viability representing IC_50_. The graph shows the mean ± SEM (*n* = 3 with four replicates each).

Since YAP/TAZ pathway as well as cellular morphology and proliferation are altered by spinach scaffold compared to conventional models, we then investigated if spinach scaffolds could also affect phenotype and drug response in melanoma SK-MEL-28 cells. Microphthalmia-associated Transcription Factor (MITF) is a lineage-determining transcription factor critical for regulation of the melanocytic lineage during development and implicated as both a melanoma tumor suppressor and oncogene (Garraway et al., 2005; Simmons et al., 2017). MITF is required for proliferation and has been identified as a factor prone to amplification (Carreira et al., 2006; Hoek et al., 2008). Two phenotypically distinct populations of melanoma cells were described related to MITF levels: high−MITF population is associated with differentiation and proliferation, whereas low−MITF cells, although they proliferate slowly, are endowed with the invasive and EMT−like characteristics (Vlèková et al., 2018). MITF is amplified in SKMel-28 cells (Wellbrock et al., 2008) and, therefore, we investigated if its expression could be altered by leaf scaffold. Immunofluorescence showed that MITF expression was upregulated in cells cultured on stiff substrate compared to leaf scaffold (Figure 4A). The number of MITF-positive cells was significantly lower (*p* < 0.016) for cells cultured on spinach leaf scaffold (Figure 4B). Gene expression was then analyzed by qRT-PCR for MITF and three genes of the MITF-high expression signature (SOX10, MLANA and TYR) (Ennen et al., 2017). Results showed that MITF, and its target genes SOX10 and MLANA were significantly down-regulated when cells were cultured on decellularized spinach leaves compared to stiff substrate, suggesting that the leaf scaffold altered MITF and its associated pathways (Figure 4C). Since MITF signature can impact drug response (Müller et al., 2014; Aida et al., 2017), we then investigated response of SK-MEL-28 to withaferin A (WFA). WFA is a natural compound from the withanolide family that induces apoptosis in human melanoma cell lines (Mayola et al., 2011) and decreases uveal melanoma tumor growth *in vivo* (Samadi et al., 2012). Dose-response curves extracted from MTT assay showed that SK-MEL-28 cells seeded on stiff substrate were more sensitive to WFA (IC_50_ = 1.1 μM) than cells on leaf scaffold (IC_50_ = 5.2 μM) (Figure 4D).

### Response of PC3 Cells to Radiation Differs Between Cells Seeded on Plastic and Leaf Substrate

**FIGURE 5:**
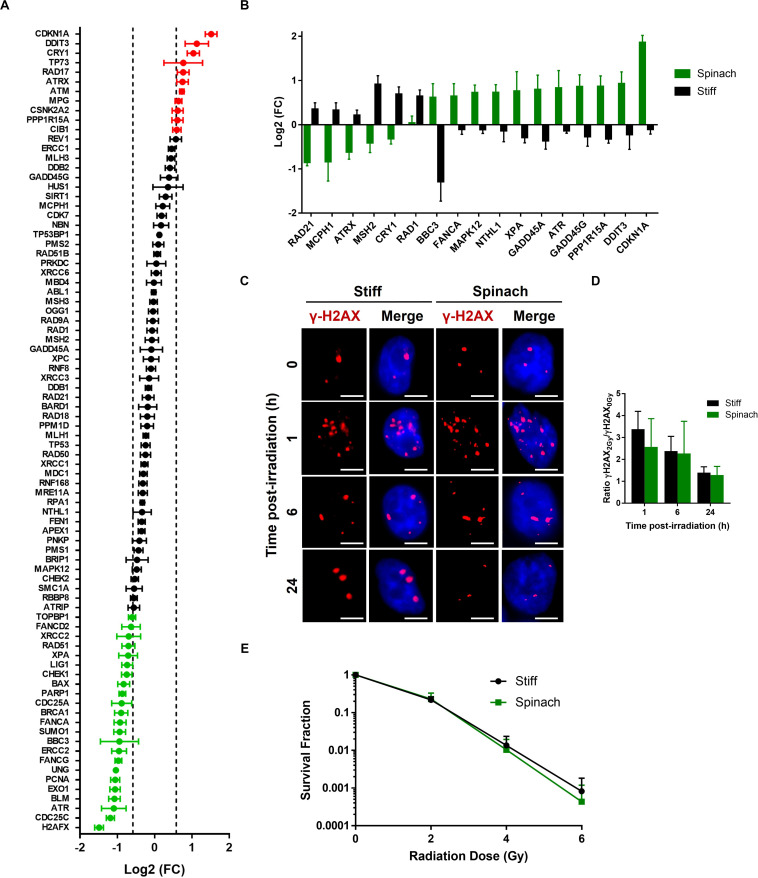
Radiation response of PC3 cells cultured on decellularized spinach leaf. **(A)** Graph showing differentially expressed genes between PC3 cells seeded on the leaf and TCPS. A total of 84 genes values have been measured (including black, red and green dots). 35 genes have | log2(FC)| > 1.5 and *p*-value < 0.05 (up-/downregulated genes, red/green dots, respectively). Data points are represented as the mean ± SEM (*n* = 3). **(B)** Graph showing genes that are differentially expressed between PC3 cells seeded on the leaf and TCPS at 6 h after 2 Gy-irradiation. Data points are represented as the mean ± SEM (*n* = 3). **(C)** Representative nuclei of PC3 cells cultured on leaf with γ-H2AX foci at 1, 6, and 24 h after 2 Gy irradiation (*n* = 3). Scale bar = 6 μm. **(D)** Histograms showing the ratio of number of 2 Gy-irradiated γH2AX foci normalized to sham-irradiated samples, in PC3 cells seeded on spinach leaf or stiff substrate (coverslip). Data points are represented as the mean ± SEM of at least 100 nuclei (*n* = 3). **(E)** Cell survival curves of PC3 cells cultured on leaf and in TCPS as assayed by colony-forming ability. Colonies with more than 50 cells were scored. Data are represented as mean ± SEM (*n* = 3).

In addition to SK-MEL-28 drug response, PC3 cells were irradiated to assess whether cell culture on leaf scaffold could also modify radiation response. We first compared the expression of 84 genes involved in DNA damage signaling pathways in PC3 cells seeded for 3 days on leaf scaffold or TCPS. Results showed that 34 of 84 genes are differentially expressed (1.5 < FC < 0.66) between the two conditions, with 11 upregulated and 23 downregulated genes in PC3 cells cultured on spinach leaf scaffold compared to cells cultured in TCPS (Figure 5A). Interestingly, the comparison of genes differentially expressed after 2Gy-irradiation revealed that 11 genes (CDKN1A, DDIT3, PPP1R15A, GADD45G, ATR, GADD45A, XPA, NTHL1, MAPK12, FANCA, and BBC3) are up-regulated and 6 genes (RAD1, CRY1, MSH2, ATRX, MCPH1, and RAD21) are down-regulated when cells were irradiated on spinach leaf scaffold (Figure 5B). p53 signaling pathway is enriched by the upregulated genes while cell cycle is enriched by the downregulated genes, suggesting a potential switch in radiation-induced DNA damage signaling between leaf scaffold and stiff substrate. In order to investigate if radiosensitivity could be affected we then assessed DNA double-strand breaks (DSBs) by monitoring the formation of γH2AX foci. As shown on Figure 5C, the number of γH2AX foci increased 1 h after 2 Gy-irradiation compared to sham-irradiated in both cells seeded on spinach scaffold or stiff substrate. At 6 h post-irradiation, the number of foci started to decrease to return to normal level at 24 h. Interestingly, the kinetic of DBSs restauration was similar between cells seeded on spinach scaffold or stiff substrate suggesting that DNA damages were effectively repaired in both conditions (Figure 5D). This was confirmed by clonogenic assay that showed that survival fraction of irradiated PC3 cells seeded on spinach leaf scaffold decreased following the same trend as cells seeded on stiff substrate demonstrating that PC3 radiosensitivity was similar between both conditions (Figure 5E).

## Discussion

The decellularization of plants to provide sustainable scaffolds that can be repopulated with human cells has gained more attention these recent years. Therefore, there is a need to better understand the cellular response that may result from this interaction. In this study, we showed that YAP/TAZ mechanotransduction pathway, cellular morphology and proliferation as well as response to drug and radiation exposure of cancer cells seeded on spinach leaf scaffolds were modified compared to TCPS or glass coverslip. Decellularized spinach scaffold display a lower stiffness (∼20 kPa) than standard cell culture substrate such as TCPS flasks or glass coverslips (∼GPa) (Seal et al., 2001; Gilbert et al., 2010) and the results we observed are in line with cellular changes observed under such circumstances. Indeed, YAP/TAZ is a known sensor for mechanical stimuli including substrate stiffness. YAP/TAZ activation leads to a nuclear accumulation YAP/TAZ and an increase of YAP/TAZ regulated genes expression that has been associated with rigid substrate (Dupont et al., 2011). Yet, YAP/TAZ activity is downregulated and mainly cytoplasmic in cells cultured on spinach leaves. Substrate stiffness is also known to influence cell morphology (Zhang et al., 2016; Xie et al., 2018) to minimize the total free energy in the cell/substrate system (Chiang et al., 2013). Our results, showing that the shape of cells cultured on spinach scaffolds adopted a round shape and diffuse actin cytoskeleton, correlate with these observations as well as studies that showed that YAP/TAZ activation and nuclear accumulation is also dictated by cell shape (Wada et al., 2011). When cell morphology is manipulated into a round and compact shape, YAP/TAZ is excluded from the nucleus whereas nuclear accumulation is observed when cells spread with the formation of F-actin stress fibers (Piccolo et al., 2014). Interestingly, the round cellular shape and the consequential transcriptional inactivity of YAP/TAZ on leaf may also explain the slow cellular growth rate compared to stiff substrate that we observed. Indeed, YAP/TAZ also stimulates cell proliferation by controlling the expression of a broad number of cell cycle regulators, of factors involved in DNA duplication and DNA repair, and of factors involved in mitosis (Zhao et al., 2007; Zanconato et al., 2015, 2019). Moreover, a soft substrate is known to decrease cellular proliferation rate (Mih et al., 2012; Liu et al., 2018). Although these cellular changes seemed to result mainly from the stiffness difference between the two models, we used a collagen and fibronectin coating to facilitate cell attachment on the leaf scaffold and we cannot exclude this did not also affect cell behavior. To evaluate this point, we compared coated and non-coated TCPS and coverslips and showed that nuclear YAP location, YAP/TAZ protein level, cellular morphology and proliferation are unchanged between these two models (Supplementary Figure S3) suggesting that the role of coating was minimal in the context of this study and that the differences between coated stiff substrate and leaf were similar to what with we observed with non-coated TCPS and coverslips. This was confirmed by a recent study that showed that biofunctionalization was not required to promote cell contractility on decellularized spinach scaffolds and that no differences in contraction were found between coated leaves, coated TCPS, non-coated leaves, or non-coated TCPS at day 7 and 21 (Robbins et al., 2020). Cell culture on such scaffolds also modified cell response to external stimuli such as drug and radiation exposure. The phenotype of SK-MEL-28 melanoma cells was altered as MITF expression was decreased. Interestingly, the relationship between MITF and stiffness have been established in a recent study that showed that collagen stiffness induces melanoma differentiation through a YAP/PAX3/MITF axis, revealing a distinct lineage-specific route of YAP signaling that contributes to the regulation of melanoma progression (Miskolczi et al., 2018). However, the role of MITF in melanoma development and progression is equivocal. For example, high levels of MITF have been reported to block proliferation (Carreira et al., 2005; Loercher et al., 2005) and its suppression improves the sensitivity of melanoma cells to a BRAF inhibitor (Aida et al., 2017), while in contrast a low MITF level promotes invasion (Carreira et al., 2006) and early resistance (Müller et al., 2014; Pathria et al., 2016). Our results showed that SK-MEL-28 cells cultured on the leaf scaffold, with a lower MITF level, are resistant to WFA. However, these results cannot be used to extrapolate a leaf scaffold-induced drug resistance, additional drug screening, with different molecular mechanisms, would be required. Overall, it is now well-known that matrix stiffness and mechanical properties influence response to drug exposure (Leight et al., 2017; Singh et al., 2018; Stylianopoulos et al., 2018) and this has to be remembered to use leaf scaffolds as tissue model for drug screening. Similarly, we showed in Figure 5A that expression of genes involved in DNA damage signaling pathways was modified when the cells were seeded on spinach scaffold compared to stiff substrate. Genes with a transcriptional regulation by p53 (ATM, CDKN1A, CSNK2A2, TP73, and RAD17) are upregulated when cells are on spinach scaffold whereas genes involved in excision repair pathways (ERCC2, LIG1, PARP1, PCNA, UNG, and XPA) are downregulated, suggesting that different repair pathways may be activated depending on the substrate stiffness. Although the cellular radiosensitivity seemed to not be affected, this alteration may lead to specific cell response to radiation under certain conditions. Unfortunately, although several studies investigated the effect of radiation on substrate stiffness, few of them extensively studied the effect of mechanical cue on radiation response. However, several studies showed that YAP promotes radioresistance and genomic instability in medulloblastoma through IGF2-mediated Akt activation (Fernandez-L et al., 2012), and conversely, its inhibition sensitizes lung cancer cells to radiation (Cheng et al., 2016). Our results do not correlate with these observations, however, it is important to note that such mechanisms are cell- and substrate-dependent, as demonstrated in a recent study that showed that YAP does not mediate mechanotransduction of breast cancer progression in 3D culture and *in vivo* but not in standard cell culture models (Lee et al., 2019). To note, although vegetal scaffolds display a three-dimensional structure, cells have been seeded on the leaf surface and spontaneously adopted a monolayer-like conformation (Figure 2 and Supplementary Figure S2). Consequently, the differences seen in this study cannot be explained by the presence of a 3D cellular organization.

**FIGURE 6:**
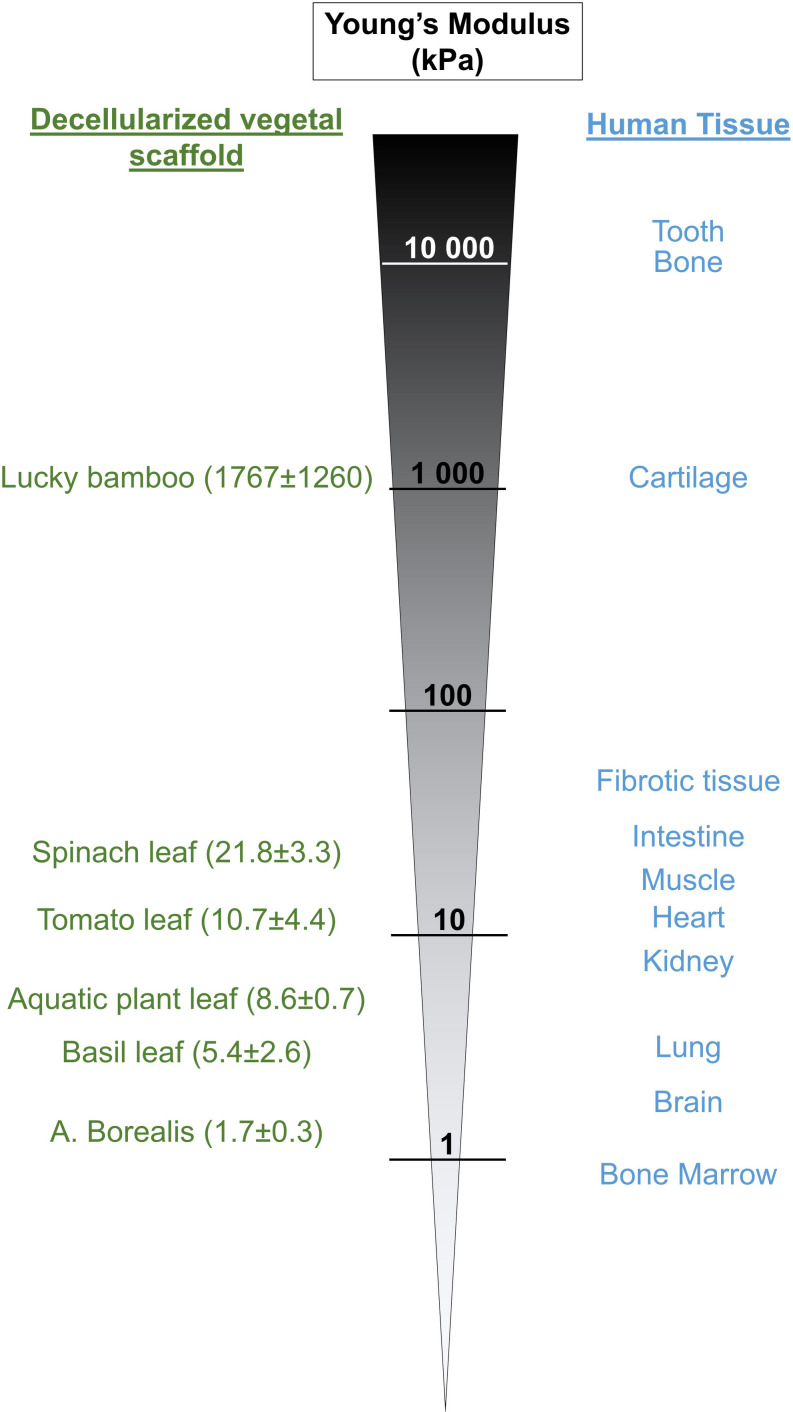
Stiffness of certain decellularized plant-based scaffolds is similar to the stiffness of human tissues. Different plant materials have been decellularized, the Young’s Modulus of the resulting scaffolds measured by AFM and compared to the published stiffness values of different human tissue.

Each plant specie is unique and displays a broad range of leaves strength and hardness (Read and Sanson, 2003; Wang et al., 2010), achieved primarily by cell walls consisting of strong fiber-rich (cellulose) composite materials (Kitajima et al., 2016), and therefore, others suggested that plants could be good candidates to provide biomaterials reproducing mechanical and physical properties of key biological tissue (Gibson et al., 2010; Hickey and Pelling, 2019). Therefore, we also assessed the stiffness of different decellularized plants, including spinach, tomato, aquatic plant, basil, A. Borealis leaves and lucky bamboo (Figure 6). Our results showed that resulting scaffolds displayed a broad range of stiffness, with Young’s modulus from 1.7 ± 0.3 (A. Borealis) to 1767 ± 1260 kPa (lucky bamboo), thus matching stiffness of main human tissues (Cox and Erler, 2011; Skardal et al., 2013; Handorf et al., 2015; Yang et al., 2017; Fekete et al., 2018) and confirming the potential use of cellulose scaffolds produced from plants decellularization to provide appropriate matrixes for reproducing the unique stiffness of specific tissue, health condition or disease progression. For example, fibrotic tissue or tumor are known to be stiffer than normal tissue (Cross et al., 2007; Liu et al., 2015), and, according to their stiffness, leaf scaffolds could be selected to mimic appropriate disease or normal microenvironment. To note that if vegetal material needs to be biofunctionalized before cell seeding, the presence of coating will significantly increase the stiffness compared to the non-functionalized decellularized scaffold (Supplementary Figure S4), offering another option to tune the scaffold stiffness accordingly (Hickey et al., 2018).

Polyacrylamide (PA) gels, polydimethylsiloxane (PDMS), polyethylene glycol (PEG), poly(methyl methacrylate) (PMMA) are currently the most commonly used substrates to investigate the effect of stiffness due to their relative ease, low price and their good reproducibility (Palchesko et al., 2012; Mullen et al., 2015; Caliari and Burdick, 2016). However, they also have limitations for a broad range of biological applications including their high hydrophobicity, their absorption of small hydrophobic molecules, their non-biodegradability or the leaching of uncrosslinked low molecular polymer to the medium (Halldorsson et al., 2015; Hoang Thi et al., 2020). In addition, they lack additional features that plant structures can provide (e.g., inner vasculature, optimal fluid transport, etc.) to complement the model and improve its accuracy.

Indeed, the architecture of vegetal scaffold is complex and rely on many different characteristics. For instance, topography can also play an important role (Jansen et al., 2015; Yang et al., 2017). The topography refers to the spatial arrangement and orientation of fibers and cells within the tissue and these features have pronounced effects on cell behavior, from cell adhesion and spreading to proliferation and differentiation. For example, the changes in tumor microenvironment topography have been showed to impact migration of immune cells (Salmon et al., 2012) and invasion of cancer cells (Levental et al., 2009). A recent study showed that cells respond to plant topographical cues by aligning with the characteristic structural patterns of plants (Fontana et al., 2017), highlighting that in addition to their specific stiffness, vegetal scaffolds could also be selected in function of their topographical features to match the desired tissue. In addition, plant-based scaffold are extremely porous matrices (Iravani and Varma, 2019). Yet several studies showed the influence of substrate porosity that can enhance cell-cell interactions and may promote tissue formation (Casillo et al., 2017) or alter mesenchymal stem cells behavior (Haugh et al., 2018). Finally, the leaf veins provide a suitable scaffold to mimic different types of vasculature architecture. Several studies showed the possibility to engraft endothelial cells within the leaf vasculature (Gershlak et al., 2017) and that these cells are even able to promote angiogenesis in chick chorioallantoic membrane assay (Dikici et al., 2019) revealing the potential of using plant scaffold to promote neovascularization for tissue engineering constructs. In addition, most cells are found no more than 100–200 μm from the nearest capillary, with this spacing providing sufficient diffusion of oxygen, nutrients, and waste products to support and maintain viable tissue (Jain et al., 2005; Lovett et al., 2009). By exploiting the minimal scale of leaf nerves and with the possibility of creating a vascularization coupled to cell culture on the leaf surface, plant-based models would offer a tissue construct allowing functional endothelium that could provide oxygen and nutriments to the generated tissue more reliably than current 3D-printed or microfluidics devices that may lack this 100–200 μm resolution.

Altogether these observations highlight the great interest of vegetal scaffolds to serve as platform to model the complex biophysical environment of specific tissue. However, they also suggest that the difference in cell behavior we observed in this study can also be due to these features, or a combination of them, and does not only rely on stiffness change. Moreover, biochemical and biomechanical composition variations of the scaffold can occur due to the intrinsic properties of the native specimen itself and to the variety of conditions in the decellularization/recellularization processes. Development of standardized protocols and characterization of each decellularized scaffolds should be considered when selecting a specific specimen to investigate biological outcomes on a targeted tissue.

The use of cancer cell lines for developing and characterizing new cellular models is often accepted due to their ease of handling and culturing, and we chose for this study two cell lines widely used for drug and radiation studies, as first line treatment for melanoma and prostate cancer are mainly chemo- and radiotherapy, respectively. However, since plant-models are gaining more importance in tissue engineering (Iravani and Varma, 2019; Jahangirian et al., 2019), the response of normal and primary cells seeded on such scaffolds to different stimuli should also be performed. Another limitation of our study is the lack of comparison with animal tissue that prevent us to conclude whether plant-based scaffolds actually reproduce more accurately the *in vivo* environment than standard models. We showed that the stiffness of plant scaffolds reproduces the stiffness of most human tissues. However, other approaches (i.e., hydrogel) can also reproduce a large range of stiffness and the advantages of decellularized plants, and their specific features (topography, vasculature, etc.), over such models would also need to be assessed. Comparison with such models would have provided important information allowing us to conclude if the difference in cellular response is uniquely due to the scaffold stiffness or to the other leaf characteristics. Finally, our study focused only on the cellular response on spinach scaffolds. Because of the high complexity and diversity of vegetal kingdom, several plants material should also be investigated since cell behavior will likely change based on the different architecture and mechanical and biochemical composition of the scaffolds.

## Conclusion

The recent emergence of decellularized vegetal material for tissue engineering and biomedical research requires a comprehensive characterization of the resulting cellulose scaffolds and their effects on the repopulated animal cells. In this study, we showed that plant decellularization provide soft scaffolds that match the stiffness range of most of the human tissue and modify cell behavior, including drug and radiation response, compared to standard cell culture models. Because of their distinguished features (natural vasculature, low immunogenicity, low cost, relative ease, etc.) and their wide variations in the shape and structures, these scaffolds offer a multi-controllable model with multiple biochemical and biophysical interactions. However, additional studies are required to determine if they could address important architectural and physical challenges of the *in vivo* tissue environment.

## Data Availability Statement

All datasets presented in this study are included in the article/Supplementary Material.

## Author Contributions

JL, AH, and FZ conceived, designed the experiments, and revised the manuscript. JL, AH, RZ, and SK conducted the experiments and analyzed the data. JL and AH drafted the manuscript. All authors read and approved the final manuscript.

## Conflict of Interest

The authors declare that the research was conducted in the absence of any commercial or financial relationships that could be construed as a potential conflict of interest.
