# Supplementary material for: Plant-Based Scaffolds Modify Cellular Response to Drug and Radiation Exposure Compared to Standard Cell Culture Models
Source: Front Bioeng Biotechnol. 2020 Aug 7;8:932. doi: 10.3389/fbioe.2020.00932 (PMC7426640; doi:10.3389/fbioe.2020.00932)
Supplement: Supplementary file 1 [file Data_Sheet_1.pdf]

Supplementary Material

Supplementary Figures

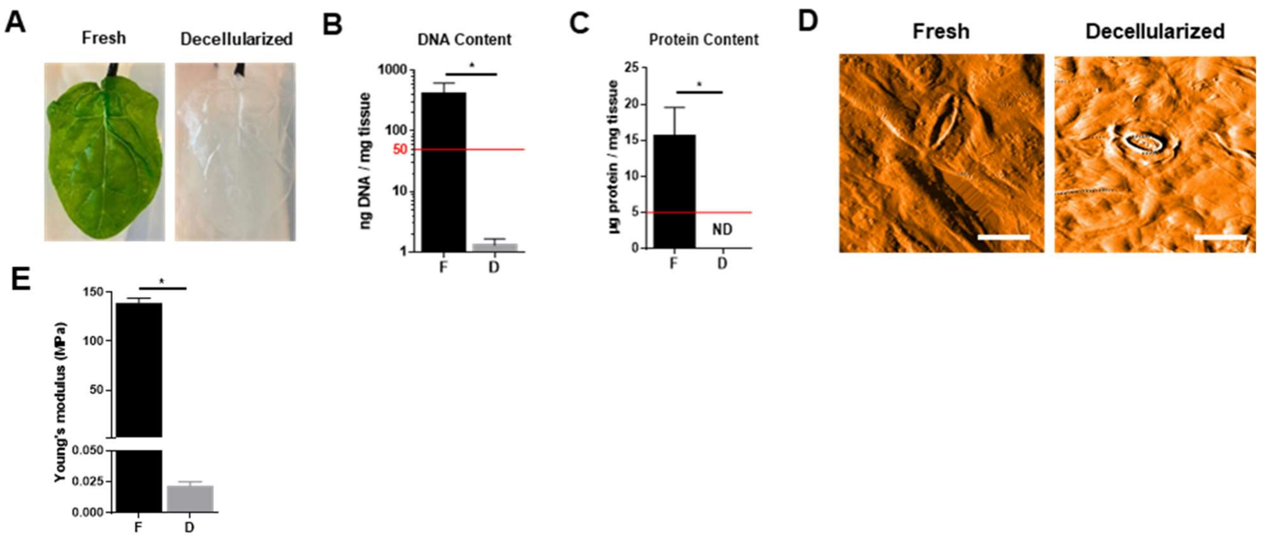

Supplementary Figure 1. Spinach leaves were efficiently decellularized. (**A**) Images of spinach leaf before and after decellularization treatment. (**B**) Comparison of DNA content in different fresh and decellularized plants represented as mean ± SEM (n=3). Red line represents the maximal amount of DNA content (50ng) per mg of tissue to consider the material decellularized. F, Fresh; D, Decellularized (**C**) Comparison of protein content in different fresh and decellularized plants quantified by micro BCA protein assay represented as mean ± SEM (n=3). Red line represents the maximal amount of protein content (5µg) per mg of tissue to consider the material decellularized. F, Fresh; D, Decellularized; ND, Not Detected (**D**) AFM images showing surface of plant materials before and after decellularization treatment. Scale bar = 20µm. (**E**) Comparison of the Young's modulus between fresh (F) and decellularized (D) spinach leaves represented as mean ± SEM. (n=3, *p<0.05; Student t-test)

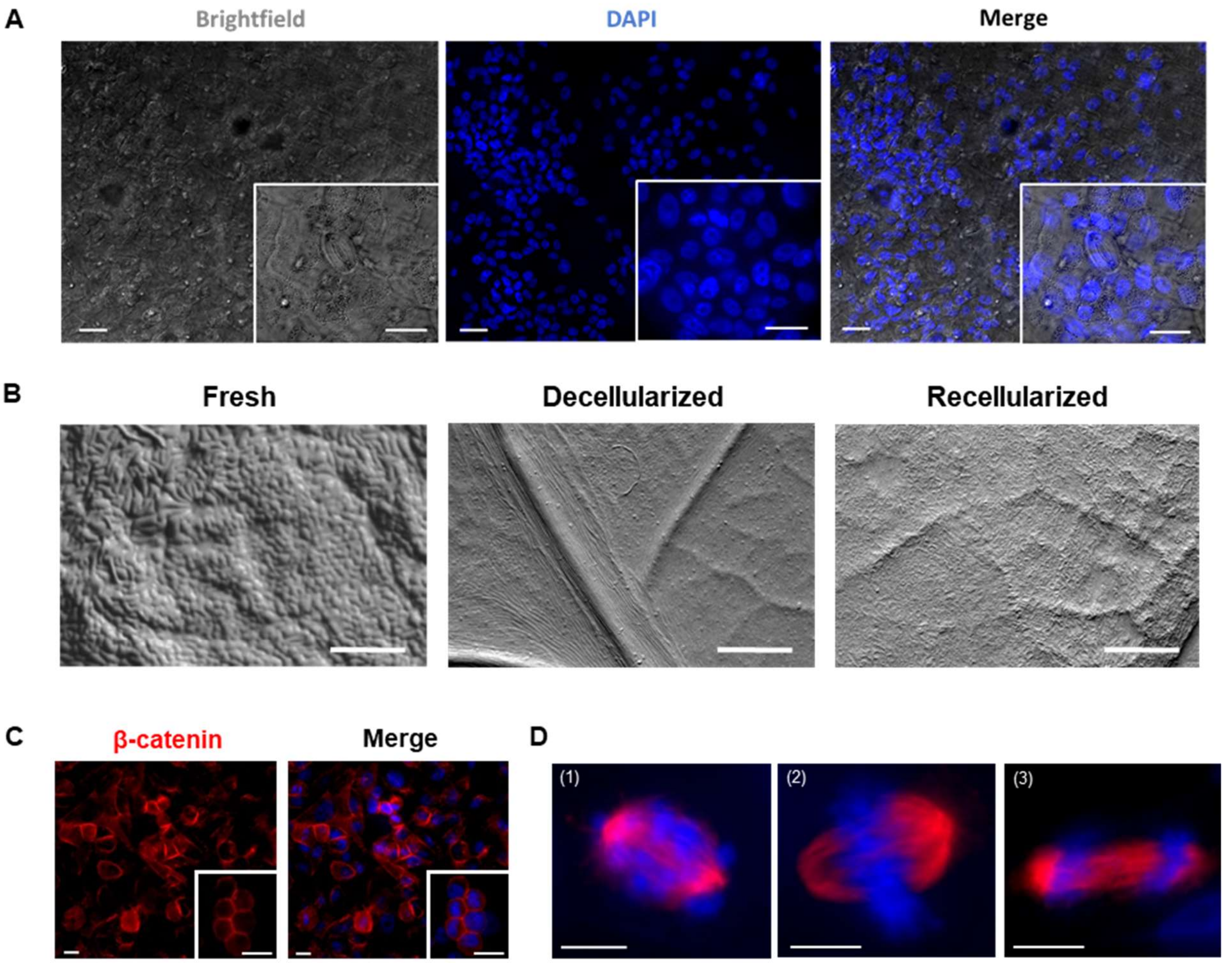

Supplementary Figure 2. PC3 cell attachment on decellularized spinach leaf scaffold. (A) Immunofluorescence images of PC3 nuclei (DAPI) seeded on spinach scaffold (brightfield). Scale bars = 10 µm. (B) 3D surface mapping of fresh, decellularized and recellularized spinach leaf by using gel-based photometric stereo profilometry. Scale bars = 500µm. (C) Visualization of β-catenin (red) by fluorescence microscopy. Nuclei have been counterstained with DAPI (blue). Scale bars = 25µm. (D) Immunofluorescence images of α-tubulin (red) and nuclei (DAPI) of PC3 cells seeded on spinach scaffold showing the presence of cells in different phases of mitosis such as prometaphase (1), metaphase (2) and late anaphase (3) Scale bars = 10µm.

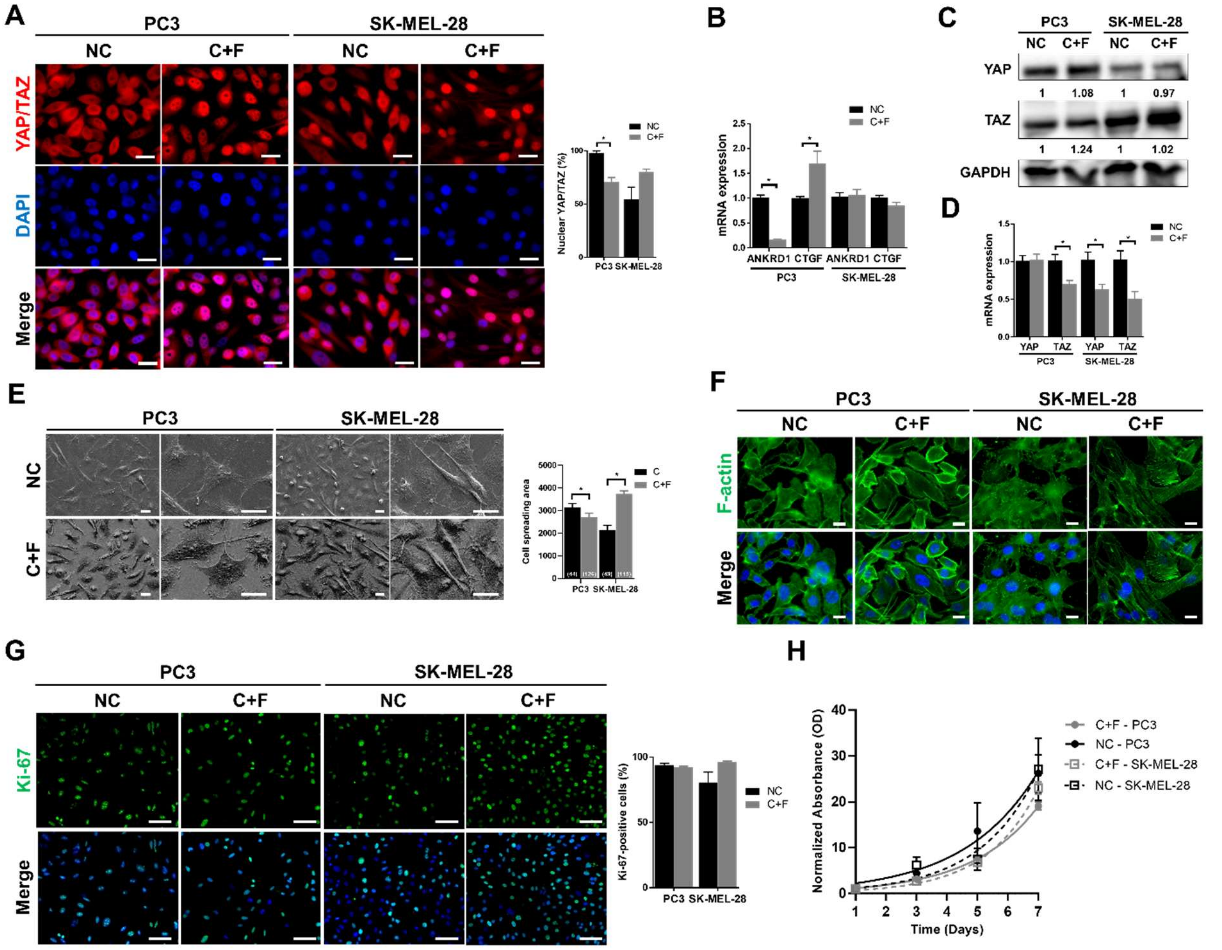

Supplementary Figure 3. Comparison of YAP/TAZ pathway, cellular morphology and proliferation between coated (C+F, collagen + fibronectin) and non-coated (NC) TCPS and glass coverslips. (**A**) Immunofluorescence images of YAP/TAZ and nuclei (DAPI) in PC3 and SK-MEL-28 cells. Scale bars = 15 µm. Graphs indicate the percentage of cells with nuclear YAP/TAZ (n=3, *p<0.05; Student t-test) (**B**) qRT-PCR for YAP/TAZ target genes (CTGF and ANKRD1) in PC3 and SK-MEL-28 cells. Data were normalized to expression on non-coated TCPS and indicated as mean ± SEM (n=3, *p<0.05; Student t-test). (**C**) Immunoblotting of YAP and TAZ in PC3 and SK-MEL-28 cells. Bands intensities were quantified using ImageJ and normalized with GAPDH. Numbers represent the expression level compared to non-coated TCPS of two independent experiments. (**D**) qRT-PCR analysis in PC3 and SK-MEL-28 cells to measure YAP and TAZ mRNA levels. Data were normalized to expression on non-coated TCPS and indicated as mean ± SEM (n=3, *p<0.05; Student t-test). (**E**) Representative SEM scanning images of PC3 and SK-MEL-28 cells cultured on NC and C+F coverslips for 3 days. The cell images were collected in three independent experiments (n=3). Scale bars = 20 µm. Histogram showing the changes of cell spreading area from (E) and represented as mean ± SEM (n=3, *p < 0.05; Mann-Whitney test). The numbers shown in parenthesis indicated cell numbers for statistics of cell spreading area examined in each case. (**F**) Immunofluorescence images of F-actin and nuclei (DAPI) in PC3 and SK-MEL-28 cells seeded on the different substrates. Scale bars = 15 µm. (**G**) Immunofluorescence images of Ki-67 and nuclei (DAPI) in PC3 and SK-MEL-28 cells. Scale bars = 60 µm. Histogram showing the percentage of Ki-67-positive cells from (G) and represented as

mean ± SEM (n=3, *$p < 0.05$; Mann-Whitney test). (**H**) Cell proliferation of PC3 and SK-MEL-28 cells seeded for 7 days on NC and C+F substrate and measured by modified MTT. Absorbance (570 nm) values were normalized from 100% at day 1 and analyzed using a nonlinear regression using exponential growth curve. Data points represent the mean ± SEM (n=3 with four replicates each).

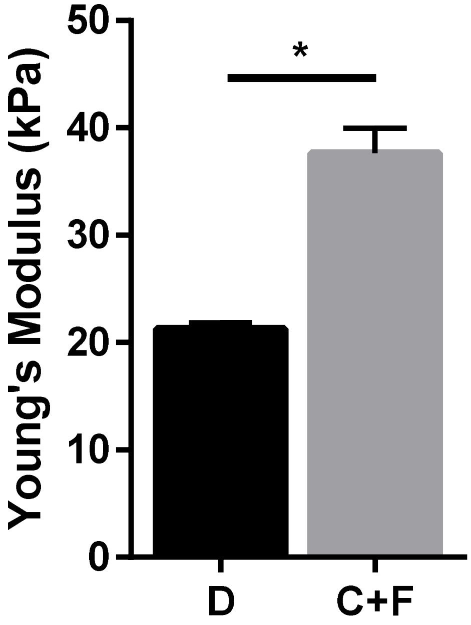

Supplementary Figure 4. Functionalization increases stiffness of decellularized scaffold. Comparison of the Young's modulus of decellularized (D) and functionalized (C+F) spinach leaf with collagen and fibronectin as measured by AFM. The graph shows the mean ± SEM (n=3, *p<0.05; Student t-test).

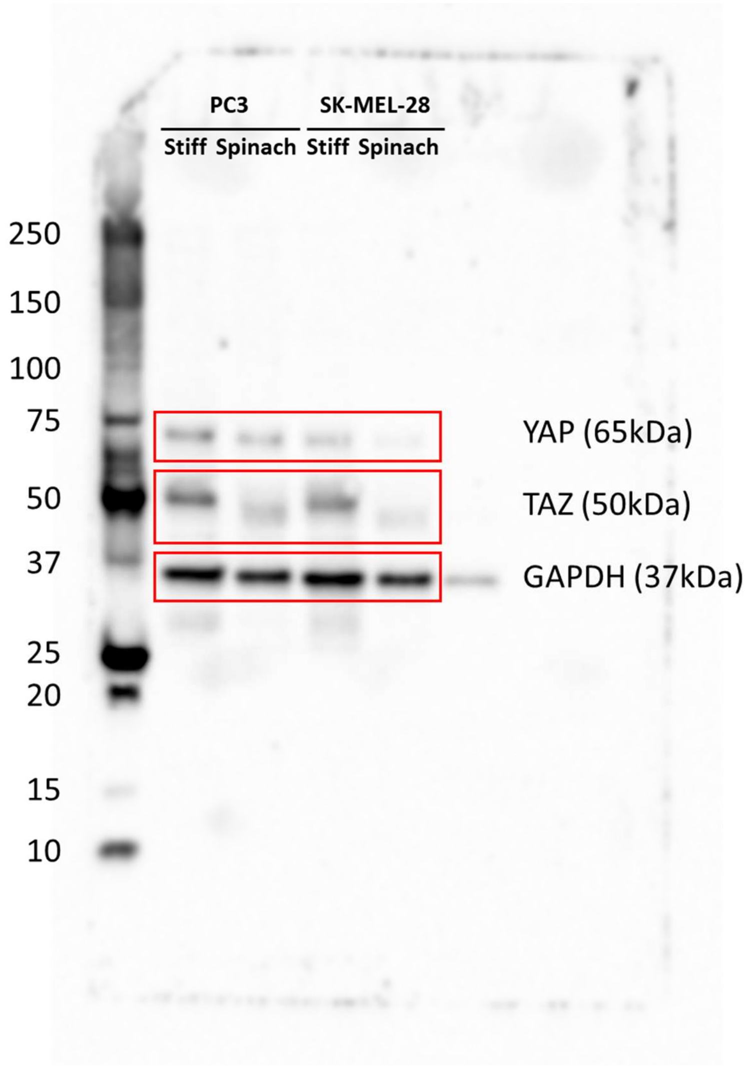

Supplementary Figure 5. Original uncropped western blot image. Red rectangles correspond to the cropped sections shown in Figure 1C.
